# Patient perspectives of using reproductive autonomy to measure quality of care: a qualitative study

**DOI:** 10.1186/s12905-023-02804-3

**Published:** 2023-12-05

**Authors:** Meredith G. Manze, Silpa Srinivasulu, Heidi E. Jones

**Affiliations:** City University of New York, Graduate School of Public Health & Health Policy, 55 W 125th Street, New York, NY 10027 USA

**Keywords:** Quality, Metrics, Reproductive health, Contraception, Pregnancy

## Abstract

**Background:**

Current measures of reproductive health care quality, such as rates of “unintended” pregnancies, neglect to incorporate patients’ desires and center their reproductive autonomy. This study explores patients’ perspectives on and receptivity to alternative metrics for measuring quality of such care.

**Methods:**

An online research recruitment firm identified eligible participants living in New York, ages 18–45, self-identifying as women, and having visited a primary care provider in the last year. We conducted five virtual focus groups and eight in-depth interviews with participants (*N* = 30) in 2021. Semi-structured guides queried on ideal clinic interactions when preventing or attempting pregnancy and their perspectives on how to measure the quality of such encounters, including receptivity to using our definition of reproductive autonomy to develop one such metric: “whether the patient got the reproductive health service or counseling that they wanted to get, while having all the information about and access to their options, and not feeling forced into anything.” We employed an inductive thematic analysis.

**Results:**

Participants wanted care that was non-judgmental, respectful, and responsive to their needs and preferences. For pregnancy prevention, many preferred unbiased information about contraceptive options to help make their own decisions. For pregnancy, many desired comprehensive information and more provider support. There was considerable support for using reproductive autonomy to measure quality of care.

**Conclusions:**

Patients had distinct desires in their preferred approach to discussions about preventing versus attempting pregnancy. Quality of reproductive health care should be measured from the patient’s perspective. Given participants’ demonstrated support, future research is needed to develop and test a new metric that assesses patients’ perceptions of reproductive autonomy during clinical encounters.

## Introduction

For decades in the United States (US), the number of “unintended” pregnancies, defined as those that are “mistimed” or “unwanted,” has been used to shape family planning programs and policies, which have invested substantial resources to reduce and prevent this perceived public health problem. For example, the Office of Population Affairs has proposed performance measures based on the percentage of women at risk of “unintended” pregnancy who are provided the most or moderately effective contraceptive methods [1].

However, aligning quality of care with such contraceptive uptake metrics risks coercing patients into using specific contraceptive methods, despite their desires around method options, and judging those who do not use contraception [2, 3]. Stigmatization of “unintended” pregnancy is further compounded by historical societal biases around who is deemed fit to parent, with low-income people, people of color, adolescents, those with substance use disorders, and people with disabilities often judged especially harshly for “unintended” pregnancy [2, 4, 5]. Such groups have been disproportionately targeted and harmed by family planning programs, due to high rates of “unintended” pregnancy and biases surrounding their reproduction [4–7]. These communities have been the central focus of research and prevention efforts—attention that often problematizes their pregnancies and devalues their “worthiness” as potential parents.

As “unintended” pregnancy constructs fail to capture meaningful elements of patients’ reproductive health needs, experiences, and care, experts urge the exploration of metrics to measure the quality of reproductive health care in ways that assess how service delivery enhances or constrains reproductive autonomy [3, 8]. Such an approach moves away from stigmatizing individuals for pregnancy and toward accountability among providers and health systems for delivering high-quality care for all [3]. As such, researchers have begun to explore alternative potential measures [3, 9, 10]. The Institute of Medicine names patient-centeredness as one key domain of quality care, defined as, “care that is respectful of and responsive to individual patient preferences, needs, and values and [ensures] that patient values guide all clinical decisions” [11]. One group developed a patient-centered contraceptive counseling measure focusing on patient-provider communication [10]. Another created a contraceptive autonomy indicator to measure whether a person has factors in place to decide and realize their preferred method [12]. Another developed and validated a 14-item *Reproductive Autonomy Scale* to measure women’s achievement of reproductive intentions with three domains related to their partner: freedom from coercion, communication, and decision-making [13].

However, none have explored what patients themselves perceive as high-quality reproductive health care delivery in a clinical encounter, broadly, and their receptivity to various metrics to evaluate quality. Primary care settings offer an opportunity for expanded provision of reproductive health services, especially critical given the recent, pervasive abortion restrictions [14].

Inclusion of patient perspectives when altering clinical practices can support their successful implementation, improve outcomes, and enhance health equity. Thus, this formative qualitative study explores New York State patients’ perspectives on and receptivity to metrics for measuring reproductive health service quality in primary care visits.

## Methods

### Study design & data collection

From October to December 2021, we recruited a purposive sample via a third-party research recruitment firm, to participate in virtual focus groups (FG) and later in-depth interviews (IDI). Eligibility criteria included self-identified women currently living in New York State, who could speak and read English, between ages 18–45, and had seen a primary care provider in the past year. We divided participants into focus groups using three age categories: 18–25, 26–35, and 36–45, to capture the unique reproductive health service needs and desires of women at different stages across the life course. The recruitment firm established quotas and emailed the screener to 80 participants within each age category. We contacted 228 eligible participants in waves; 45 individuals agreed to participate and, of those, 30 were enrolled.

The semi-structured FG guide domains centered on experiences and perceptions of what makes for a good primary care visit generally and for reproductive health care, an ideal visit when seeking information or services on preventing pregnancy and for having a healthy pregnancy, and their receptivity to using a proposed definition of reproductive autonomy to measure quality of reproductive health service delivery. Results are reported elsewhere on their perspectives on telehealth visits and providers asking an open-ended reproductive health service needs question (versus pregnancy intentions question) [15, 16]. We queried specifically for feedback on using reproductive autonomy as a measure of quality of care. We defined this as, “whether the patient got the reproductive health service or counseling that they wanted to get, while having all the information about and access to their options, and not feeling forced into anything.” This definition combined the notions of reproductive autonomy with Senderowicz’ contraceptive autonomy, to operationalize as a metric [3, 12, 13]. The definition was screen-shared during data collection. We also asked when and how they would prefer to provide feedback on the quality of their clinic visit. The research team iteratively refined the FG guide after each session. FGs lasted 50 min to two hours, with only participants and researchers present. We discussed the informed consent document and the goal of the study, to inform practice guidelines and discussions about reproductive health, and obtained verbal consent prior to audio-recording.

Following each FG, the co-moderators (MM, SS) and co-Principal Investigator (HJ) practiced reflexivity and discussed emergent themes, personal biases, and strategies for improving FG moderation. We individually journaled in a shared document. Through discussion, we determined that we did not reach conceptual saturation among age groups 18–25 and 26–35 due to scheduling and participation challenges [17]. Therefore, we repeated recruitment for these ages and transitioned to conducting IDIs. We adapted the FG guide into a semi-structured interview guide. Interviews lasted 25–60 min. We conducted five FGs (two each with 18–25 and 36–45 year-olds; one with 26–25 year-olds) and eight IDIs (four each with 18–25 and 26–35 year-olds). Thirty participants were included in this study (22 in FGs and eight IDIs).

Immediately following each FG and IDI, we sent participants a brief online survey. Participants received $30 within 24 hours of completing the survey. Audio recordings were professionally transcribed. Participants did not review transcripts nor provide feedback on the findings. All participants used a pseudonym for on-screen names and as reported in this study.

### Analysis

We employed inductive thematic analysis to analyze perceptions of quality metrics for reproductive health services. The analytic team (SS, MM, HJ) identify as cis women, with divergent experiences of having biological, non-biological, and no children, and are significantly involved in reproductive health research in primary care settings and support expanded access to such services. The analysts began by independently reading three FGs, employing block coding, and writing memos. We reviewed coding, reconciled discrepancies, and refined the codebook. One author (SS) coded all remaining transcripts; another (MM) functioned as a second coder for the IDIs she did not conduct, to ensure both were familiar with all data. The final codebook was organized within the following high-level codes related to sexual and reproductive health (SRH): discussion desires, history of care, measuring quality of care, telehealth, and services needs approach (in clinical encounter). Both took extensive memos throughout the coding process to document emerging themes and differences across age groups. They first used memos and field notes to identify key themes, make connections, and generate initial theory; then, they reviewed and sorted coded excerpts to deepen the analysis. We utilized Dedoose version 9.0.17 (Los Angeles, CA) to manage data.

This study adheres to guidelines stated in the Consolidated Criteria for Reporting Qualitative Research. The Institutional Review Board of the City University of New York approved this study.

## Results

### Sociodemographic characteristics

Of the 30 participants, most (60%) were from New York City, identified as people of color (60%), and felt they had a regular health care provider (83%). Over three-quarters (77%) had completed some college or higher. Half were single, and nearly half (47%) were married or in a committed relationship. Most participants had only had sex with men (67%), six (20%) with only women, and three (10%) with both genders. Most (60%) were able to get pregnant, three (10%) self-reported that they were not able, and nine (30%) were unsure.

### Overview of thematic findings

Overall, participants wanted sexual and reproductive health care services that are non-judgmental and respectful, with a provider whom they trust and who creates a comfortable space for patients. They felt providers should ensure that patients’ questions are answered fully, without feeling rushed, and provide the services needed without excess treatment or testing.

“Being open and honest with my provider is important, so that I can get all of the information necessary to move forward in being healthy. And I would hope that my provider is receptive as well, and not limiting, and the information that they give me as well is not biased.” IDI #4, age 18-25

We asked participants what they would like to discuss with a provider, and how they feel it should be discussed, separately for preventing pregnancy and getting pregnant. For pregnancy prevention, they preferred unbiased information about contraceptive options to help them make their own decisions. For discussions about getting pregnant, they desired comprehensive information and provider input and support. Although both noted elements of collaboration, for preventing pregnancy participants perceived themselves as the ultimate decision-makers.

#### Discussions on preventing pregnancy

Participants desired clear and comprehensive information on contraception, including options for their partner. They felt providers should recognize that patients will make their own decisions about if and what contraceptive methods to use.

Participants wanted to be fully informed and for their providers to answer all their questions. However, they wanted this counseling tailored to their needs and desires, and responsive to their questions for information. This suggestion may translate into providers only answering specific questions, to being asked to share comprehensive information about contraceptive options. The following two quotes represent both ends of this spectrum:

“I’d just confirm with the doctor that like my birth control is still working, if I had any side effects, I'd bring it up, but otherwise, I wouldn't spend too much time on it honestly.” FG 1, age 18-25

“Informative. Answering every question that I have. Requesting whatever I don't know about.” IDI 1, age 26-35

Of key importance was for providers to be responsive to patients’ desires for the amount and type of information needed about contraceptive options.

Counseling should be compassionate and respectful towards patients, demonstrated by avoiding pressure to use certain contraception, respecting patients’ decisions to have or not have children, and valuing patients’ expertise: “sometimes it’s okay for a doctor to just listen to their patients, maybe hear some of their ideas…because sometimes the patients may have good ideas too” (IDI #3, age 26–35). Participants wanted to feel heard and valued for their role in contraceptive decision-making. They desired collaboration where the patient makes the ultimate decision with the support and guidance requested from their provider. For example, a younger participant shared her preferences for these conversations to center her agency to make decisions based on providers’ input and information about birth control:

“All my options. I just don't want to be told what [providers] think is best. I want to be able to decide that for myself. I want it straightforward, tell me – basically, give it to me straight. Yeah, not anything patronizing, condescending. Just, you know, give me all my options and then, we'll go from there.” IDI #5, age 18-25

In contrast, the older age group was more welcoming of provider input on contraceptive methods than the younger age groups:

“The fact that you're having that collaborative, open dialogue with your provider… you feel like your opinions are being recognized and respected and you're making choices for your body and for your health, and you want to make sure that your medical team is on board with that, and that they're in the same vein as you. Otherwise, problems rise.” FG #3, age 36-45

#### Discussions on getting pregnant

For discussions about getting pregnant, participants wanted details on discontinuing contraception, their fertility/ability to conceive, and options for having a child. They also desired information about how to prepare for a healthy pregnancy, for both baby and parents, and potential risks.

Participants across age groups wanted counseling and pregnancy services presented comprehensively, informatively, and tailored to their specific needs. Because there were more unknowns on how to prepare for pregnancy, especially among nulliparous participants, they felt providers’ roles should be more prominent in these discussions, as opposed to pregnancy prevention. One participant summarized, “I would like to feel like [providers are] with me the whole way” (FG #2, age 18–25).

Participants expressed wanting support throughout their pregnancy. Those who had specific health issues or pregnancy risks wanted more oversight and detailed information tailored to their concerns. One described, “Just going from A to Z, going over everything, having your questions answered, your doctor asking you questions” (FG #3, age 36–45).

Like pregnancy prevention discussions, participants felt counseling and services for getting pregnant should be offered in non-judgmental, unbiased, and respectful ways. Several participants acknowledged that their providers assumed that women would want children when they are a certain age. Similarly, one participant noted the assumptions her provider made in that she would not want more children because she had three. When providers have made such assumptions, they were perceived as judgmental, disrespectful, and biased toward a certain ‘ideal’ reproductive role of women. This juxtaposed participants’ feelings of being an expert in their own care.

### General: reproductive autonomy responses

When presented with the suggestion of using our definition of reproductive autonomy to measure quality of reproductive health care, most participants supported the idea. The following feedback illustrates participant support:

Snoopy: “I think it's a good way to measure it, grade it.”

Shamika: “It's positive.”

Doris: “Yeah. You don't want to be coerced into something you don't think it's beneficial to you, such as a hysterectomy.”

Snoopy: “But you want to be informed about your option of having that or whatever you may need.”

Ruby Bird: “Exactly…. that seems kind of like a good summary of basically everything that we've all been saying over the last hour or so.” (FG #6, age 36-45)

However, a few felt the label “reproductive autonomy” was overly complicated and preferred to conceptualize this simply as quality care or provision of reproductive health services. Some also noted that just because patients want a service or medication does not necessarily mean they should have access, for example, if it is not clinically indicated.

### How to capture feedback

Most participants preferred sharing feedback or measuring their provider’s quality of care through email or text message after the visit to allow them time to process their feedback. Several highlighted that they would want to know if and how feedback would be used to improve practice; this would serve as motivation to complete the post-visit assessment. One participant shared:

“If that feedback is going to get to the doctor, is it really going to get to the point where he's going to make a change?... So, I'm not really sure if they're gonna make things happen, it's like you give your feedback, but is there anything that's going to change?” (FG #2, age 26-35)

## Discussion

Our findings demonstrate that participants support the notion that quality of reproductive health services should measure *how,* not only *what,* care is provided, as many current measures do. Participants’ perceptions of quality metrics reflected principles of reproductive autonomy as defined in this study and in other research: tailored, respectful, compassionate care that enables trust in one’s provider and empowers patients to exercise their own power, autonomy, and decision-making over their reproduction [3, 12, 18, 19]. However, their preferences for ideal, high-quality counseling approaches for preventing pregnancy versus having a healthy pregnancy were qualitatively distinct.

Providers’ counseling approaches should acknowledge patients’ unique reproductive health service needs and experiences. Patients trying to become pregnant for the first time may prefer guidance, support, and discussion for healthy pregnancy. However, others may prefer a more hands-off approach to pregnancy prevention counseling due to their own familiarity with the topic and seeing themselves as the expert and decision-maker in this care [20–22]. Given this preferred approach to preventing pregnancy discussions, the key to enhancing quality may be to move beyond “shared” *decision-making* and center the *mutual expertise* between the provider and patient. A shared decision-making approach often involves the provider contributing their medical knowledge and encouraging patients to contribute their own values and preferences as the experts on their lives. But central to this approach is that together they come to a decision on the patient’s care [23]. While this approach may work for some, others may not desire a rigorous back-and-forth of information sharing, values clarification, deliberation, and interactive questioning. Some patients may wish to make decisions informed by the mutual expertise of themselves and their providers.

Participants expressed support for measuring quality of reproductive health care through operationalizing our definition of reproductive autonomy [3, 12, 13]. Their responses suggested that they expect this care to inherently reflect principles of access and availability of desired reproductive health counseling and services, informative care delivery, and non-coercion.

Furthermore, participants in our study largely agree with opportunities to offer feedback anonymously after having time to process, through simple surveys. This suggests acceptability for integrating a patient-facing mechanism to collect data on a reproductive autonomy metric. Importantly, patients want the health center to explain how feedback will be used. If they do not perceive their feedback as improving the patient-provider interaction and quality of care delivery, then their desire to answer such surveys diminishes.

This study has several limitations. As our sample was limited to women living in New York from an online research recruitment panel, our findings may not reflect the perspectives of other distinct communities, including non-English speakers, older women, and other genders. Additional research is needed to understand these populations’ ideas for measuring quality of reproductive health services. While the flexibility of qualitative research methodology allows for adjusting data collection strategies, transitioning from focus groups to in-depth-interviews to address scheduling and engagement challenges among younger age groups may have affected the results generated; although, we did not identify different themes by data collection method. We were not able to stratify our sample beyond age based on socioeconomic status, parity, or other characteristics that may affect desired approaches to reproductive health service needs and receptivity to reproductive autonomy as a measure of quality of care.

## Conclusions

Our findings indicate that assessing perceptions around reproductive autonomy during clinical encounters may be a metric that aligns with patient values for measuring the quality of reproductive health care. Further research is needed to develop, validate, and test a data collection tool to operationalize this measurement. Until we develop metrics to track the quality and effectiveness of reproductive health service delivery beyond reducing “unintended” pregnancies, we will continue to blame “poor” outcomes on individuals who become pregnant, and not on structural facilitators and barriers that empower or impede individuals from exercising reproductive autonomy and leading healthy lives with dignity.

## Data Availability

The data generated and analyzed during this study are not publicly available due to the potential of breaching confidentiality.
